# MRI biomarkers for Alzheimer's disease: the impact of functional connectivity in the default mode network and structural connectivity between lobes on diagnostic accuracy

**DOI:** 10.1016/j.heliyon.2022.e08901

**Published:** 2022-02-02

**Authors:** R. Mohtasib, J. Alghamdi, A. Jobeir, A. Masawi, N. Pedrosa de Barros, T. Billiet, H. Struyfs, T.V. Phan, W. Van Hecke, A. Ribbens

**Affiliations:** a Molecular & Functional Imaging, King Faisal Specialist Hospital & Research Center, Riyadh, Saudi Arabia; b Alfaisal University, Department of Medical School, Riyadh, Saudi Arabia; c Department of Diagnostic Radiology, Faculty of Applied Medical Sciences, King Abdulaziz University, Jeddah, Saudi Arabia; d icometrix, Clinical Trials, Leuven, Belgium

**Keywords:** Alzheimer's disease (AD), Icobrain, Brain volumetry, DTI, DKI, rsfMRI

## Abstract

**Background:**

At present, clinical use of MRI in Alzheimer's disease (AD) is mostly focused on the assessment of brain atrophy, namely in the hippocampal region. Despite this, multiple biomarkers reflecting structural and functional brain connectivity changes have shown promising results in the assessment of AD. To help identify the most relevant ones that may stand a chance of being used in clinical practice, we compared multiple biomarker in terms of their value to discriminate AD from healthy controls and analyzed their age dependency.

**Methods:**

20 AD patients and 20 matched controls underwent MRI-scanning (3T GE), including T1-weighted, diffusion-MRI, and resting-state-fMRI (rsfMRI). Whole brain, white matter, gray matter, cortical gray matter and hippocampi volumes were measured using icobrain. rsfMRI between regions of the default-mode-network (DMN) was assessed using group independent-component-analysis. Median diffusivity and kurtosis were determined in gray and white-matter. DTI data was used to evaluate pairwise structural connectivity between lobar regions and the hippocampi.

Logistic-Regression and Random-Forest models were trained to classify AD-status based on, respectively different isolated features and age, and feature-groups combined with age.

**Results:**

Hippocampal features, features reflecting the functional connectivity between the medial-Pre-Frontal-Cortex (mPFC) and the posterior regions of the DMN, and structural interhemispheric frontal connectivity showed the strongest differences between AD-patients and controls. Structural interhemispheric parietal connectivity, structural connectivity between the parietal lobe and hippocampus in the right hemisphere, and mPFC-DMN-features, showed only an association with AD-status (p < 0.05) but not with age. Hippocampi volumes showed an association both with age and AD-status (p < 0.05).

Smallest-hippocampus-volume was the most discriminative feature. The best performance (accuracy:0.74, sensitivity:0.74, specificity:0.74) was obtained with an RF-model combining the best feature from each feature-group (smallest hippocampus volume, WM volume, median GM MD, lTPJ-mPFC connectivity and structural interhemispheric frontal connectivity) and age.

**Conclusions:**

Brain connectivity changes caused by AD are reflected in multiple MRI-biomarkers. Decline in both the functional DMN-connectivity and the parietal interhemispheric structural connectivity may assist sepparating healthy-aging driven changes from AD, complementing hippocampal volumes which are affected by both aging and AD.

## 1 Introduction

In clinical practice, Magnetic Resonance Imaging (MRI) helps in the decision making for the diagnosis of Alzheimer's disease (AD). Structural changes, more specifically hippocampal atrophy, are currently the main imaging criteria for supporting the diagnosis of AD, as recommended by the 2011 National Institute on Aging – Alzheimer Association criteria for Alzheimer's disease (AD) [1, 2]. However, due to the limited specificity of hippocampal atrophy for AD [3], many more structural and functional brain measures have been explored as potential biomarkers for AD diagnosis.

Functional and structural changes in the connection between different brain regions have been observed in Alzheimer's disease (AD), supporting the idea of a disconnection syndrome [4]. It is hypothesized that these alterations result, at least partially, from the progressive impairment of fiber tract connectivity and integrity [5, 6, 7]. Structural and functional brain connectivity have been studied using advanced MRI techniques and have provided better insights for understanding the biological and functional processes related to AD.

One particular field of interest is white matter (WM) integrity, which is mainly assessed through diffusion tensor imaging (DTI), a technique that provides directional information of the diffusion of water. In healthy WM, water diffuses preferentially anisotropically along the WM tracts, i.e. diffusion is mostly determined by the bundles of axons and their orientation. Consequently, damage to the integrity of the fiber tracts leads to changes in the diffusivity of water in the brain, making DTI an interesting tool to assess the integrity of WM fibers [8, 9, 10, 11]. In AD, it has been shown that the mean diffusivity of the water molecules increases, while the directionality of the water diffusivity (measured by fractional anisotropy) decreases, especially in the temporal and parietal lobes [12, 13, 14, 15, 16]. This is confirmed by the more sensitive diffusion kurtosis imaging (DKI) technique, which assesses the non-gaussian component of water diffusion [17, 18, 19].

Decreased WM integrity is hypothesized to be associated with WM degeneration. Indeed, WM volume loss in AD has been reported, with changes being associated with memory loss and increased risk of progression from mild cognitive impairment to AD dementia [20, 21, 22, 23].

Another key research field is resting-state functional imaging (rsfMRI), which extracts information regarding functional connectivity between parts of the brain during spontaneous neural activity (in absence of stimuli). The brain has several low-frequency resting-state networks, such as the default mode network (DMN), which is an interconnected and anatomically defined brain system preferentially active during the focus on internal tasks such as daydreaming [24, 25]. Multiple studies reported decreased DMN connectivity, especially in the precuneus and posterior cingulate cortex, in the AD continuum [26, 27, 28, 29, 30].

The clinical benefits of structural and functional connectivity biomarkers in measuring changes in AD have been well demonstrated [31, 32, 33, 34, 35]. Although the connectivity-based features were compared with the conventional structural changes (e.g. hippocampal volumetry) [36, 37, 38], it is still unclear which are the most relevant ones among this multitude of features to be used as additional MR biomarkers for the clinical evaluation of AD. In this work, we aim to determine the most relevant MRI features that could be used in clinical practice. First, we analyzed how the different features from T1, DTI, DKI and rsfMRI change due to the presence of AD as well as due to normal aging in order to validate previous findings on an independent dataset. To support the implementation of diffusion and rsfMRI biomarkers into clinical practice, the analysis was complemented by a comparative study of the AD/control classification performance obtained with machine learning models trained with different sets of features as well as each feature individually. In particular, we want to evaluate if combining diffusion and functional features with hippocampal volumetry can help in increasing the diagnostic certainty of AD.

## 2 Methods

### 2.1 Data

Cross-sectional brain MRI scans (i.e. single time point) including 3D T1, diffusion MRI (dMRI), and rsfMRI series were obtained from 40 subjects, including 20 AD patients and 20 cognitively healthy controls (Table 1). Controls were selected to match the AD group in terms of age and sex distribution. All datasets were acquired in King Faisal Specialist Hospital & Research Centre, on a 3T GE MEDICAL SYSTEMS DISCOVERY MR750 scanner, using a 32 channels head coil.

**Table 1:** Demographics for the AD and Control groups. P-values comparing age and sex between groups were determined, respectively, using a Welch two-sample t-test and Pearson's Chi-squared test.

	AD	Control	p-val
Nr.	20	20	
Age (mean ± SD; range)	66.7 ± 11.1; (43–77) years	62.4 ± 8.6; (48–77) years	0.18
Sex (Nr. Males (%Male))	12 (60%)	12 (60%)	1.00

Diffusion data was acquired using multi-shell HARDI (8 x b = 0, 25 x b = 700, 45 x b = 1200, 75 x b = 2800, TR 7800 ms, TE 100 ms, flip angle 90°) with phase encoding along the AP (Anterior-Posterior) direction. Datasets were complemented by 3 b = 0 images and 6 b = 2800 images acquired with phase encoding along the PA (Posterior-Anterior) direction. The voxel size of diffusion data was 2.4 × 2.4 × 2.4 mm.

The rsfMRI series contained 300 volumes, acquired with a TR of 2 s, TE 30 ms, and a flip angle of 77°. The data matrix for each volume had an isotropic resolution of 3 mm and dimensions 64 × 64 × 42 voxels.

Finally, 3D T1 MRI series were acquired with TR 7.9 ms, TE 3.06 ms, TI 450 ms, and flip angle 12°. The typical resolution of T1 images was 1.00 × 0.94 × 0.94 mm.

This study was approved by the ethical standards of the institutional and/or national research ethics committee of King Faisal Specialist Hospital & Research Centre (REF:C380/1100/41). All subjects had given informed consent according to the declaration of Helsinki.

### 2.2 Clinical and neuropsychological assessment

All patients were diagnosed according to strictly applied clinical diagnostic criteria and by consensus by at least two neurologists, experienced in neurodegenerative disorders. Diagnosis of probable AD was based on NINCDS/ADRDA criteria, with all patients fulfilling the DSM-IV-TR criteria as well [39, 40].

Cognition and behavior were assessed at inclusion, covering a period of 2 weeks prior to inclusion and using a battery of assessment scales including the Mini-Mental State Examination (MMSE) [41], Verbal Fluency Test [42], Neuropsychiatric Inventory [43] and Geriatric Depression Scale [44].

### 2.3 Brain volumetry

Normalized white matter (WM), gray matter (GM), cortical gray matter (CGM), and left and right hippocampal volumes were obtained from the cross-sectional icobrain dm pipeline, previously termed MSmetrix [45] (www.icometrix.com). The method takes a T1-weighted image as an input.

Tissue class segmentation was performed on the skull stripped T1-weighted image using an expectation-maximization algorithm. The algorithm optimizes a Gaussian mixture model on the image intensities while correcting for field inhomogeneities, guided by the probabilistic tissue priors. A spatial consistency model based on a Markov Random Field is also included in the algorithm. Finally, the volumes for whole brain (WM + GM), GM, CGM, and WM were extracted from these segmentations.

A fully automated algorithm was used to segment the hippocampus by utilizing information about the anatomical shape and hippocampus image intensities based on multiple atlases registration and label fusion approach [46, 47]. The hippocampus segmentation was further refined with post-processing steps using localized intensity information from the image.

Quality control was performed for all segmentations. All volumes were normalized to the same head size using a common reference atlas in MNI (Montreal Neurological Institute) space [48] with an intracranial volume of 1990 ml.

Finally, the volumes of the following structures were determined for each subject: CGM, GM, WM, Whole Brain (WB), left and right hippocampi. Moreover, given the importance of the hippocampal volumes, the following additional features were calculated, the volume of the smallest and largest hippocampus, total hippocampi volume, and relative hippocampal asymmetry determined as:

$$rel.\;hippoc.\;asymmetry\;\left(\%\right)=200\times \frac{\left|v_{Hippoc.}^{L}-v_{Hippoc.}^{R}\right|}{v_{Hippoc.}^{L}+v_{Hippoc.}^{R}}$$

where $v_{Hippoc.}^{L}$ and $v_{Hippoc.}^{R}$ are respectively the volumes of the left and right hippocampi.

### 2.4 Diffusion data processing

DTI/DKI data were pre-processed at icometrix using an in-house developed pipeline based on previous publications [49, 50]. This pipeline performs the following steps: denoising, Gibbs ringing artifacts removal, bias field correction, motion and eddy current-induced distortion correction, and correction for susceptibility-induced artifacts. In all registrations, the reorientation of the B-matrix was taken into account, and the resolution was optimally preserved through a single interpolation step. Next, the DTI and DKI tensors were estimated using an iteratively reweighted linear least square fitting routine, robust for outliers. From the DTI tensor, fractional anisotropy (FA), mean diffusivity (MD), radial diffusivity (RD), and axial diffusivity (AxD) were calculated. From the kurtosis tensor, axial kurtosis (AK), radial kurtosis (RK), and mean kurtosis (MK) were obtained.

The median values of the collected diffusion parameter images were determined for WM and GM. For this, the WM and GM masks, previously obtained from the structural T1 data, were registered to the preprocessed DWI datasets and median values in WM and GM were determined for DTI and DKI parameters. Given that diffusion is more isotropic in GM, the determination of the principal diffusion direction is suboptimal, leading to errors in axial and radial diffusion measures. Despite this, given that median values were determined across the whole GM and CGM, these measures are expected to be reliable. Nevertheless, in GM the main focus should be on MD and MK.

In order to extract structural connectivity features, whole brain tractograms were built using probabilistic tractography based on fiber orientation distribution obtained with constrained spherical deconvolution [51]. The total count of streamlines (n = 1 million) for whole brain reconstruction was the same in all subjects. Anatomical constraints [52] and spherical-deconvolution informed filtering [53] were also applied to obtain more biologically accurate tract reconstructions. Connectomes were then constructed by counting the number of streamlines linking pairs of regions of interest: the four major lobes and the hippocampi in both hemispheres. The number of streamlines linking pairs of regions were used as structural connectivity features (i.e., Struct. Connect. region 1 – region 2). The Brain Connectivity toolbox was used to extract four structural network features: global efficiency, characteristic path length, transitivity and mean clustering coefficient [54].

### 2.5 Resting-state fMRI data processing

rsfMRI data were corrected for motion artifacts by rigid realignment of the 4D time series, including slice timing correction. Each subject's corrected rsfMRI data were then co-registered with their anatomical data through affine registration of the mean fMRI image and the T1 weighted image. For normalization to MNI space, first, a population-specific template was constructed from the anatomical T1 data. This ensures a better registration with the MNI template. The template was created based on the data from 5 patients and 5 controls, considering the proper representation of age and gender of the whole population. The affine and nonrigid registrations from anatomical data to the population template and from the population template to the MNI template were then applied to the corrected rsfMRI data. Finally, the rsfMRI data were smoothed using a Gaussian kernel with a **f**ull width at half maximum ​of 6 mm.

**Figure 1:**
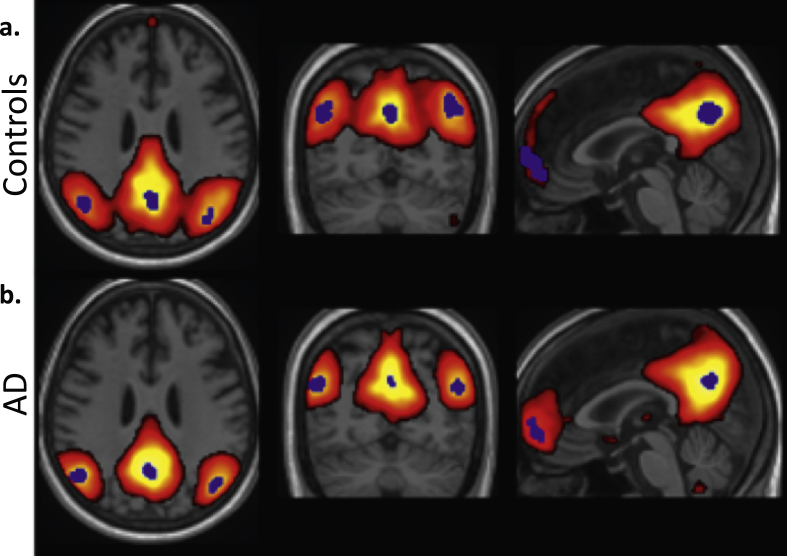
The z-transformed default mode network component from group-ICA in a) controls and b) patients separately. The blue ROIs indicate the core of the main DMN clusters, obtained by region growing starting from the local maximum.

After data pre-processing, group independent component analysis [55] was run on the group of patients and the group of controls separately, with various number of components. Based on visual inspection, the results obtained with 15 components were selected considering their ability to extract the DMN in both groups (Figure 1).

Intensity values of the DMN component were Z-transformed and a z-threshold of 2.8 was used in both groups to optimally separate the main clusters composing the DMN. The position of the local maximum was defined for the four typical DMN clusters: posterior cingulate cortex (PCC), left temporoparietal junction (lTPJ), right temporoparietal junction (rTPJ), and the medial prefrontal cortex (mPFC). Centered on these local maxima, a region growing approach was applied, resulting in equally sized regions-of-interest (ROIs) of 120 voxels. Compared to spherical ROIs, this approach ensures the ROIs represent the center of DMN functional activity more accurately, as illustrated in Figure 1. Within each ROI the participant-specific functional activity (i.e. voxel time series) was averaged, considering a band-pass filter allowing signal fluctuations between 0.01 Hz and 0.1 Hz, i.e. the typical frequencies of resting-state activity.

Functional connectivity was obtained by correlating the filtered functional time-series between each two out of four ROIs and Z-transforming the resulting Pearson correlation coefficients using the Fisher Z-transformation. As such, for each connection of each participant, a Z-score was obtained, representing the strength of connectivity. A subject's average DMN connectivity was determined as the average absolute Z-score across connections. In summary, the following DMN connectivity features were extracted for each subject: avg. DMN connectivity, and connectivity between all region pairs, i.e. PCC-lTPJ, PCC-mPFC, PCC-rTPJ, lTPJ-mPFC, lTPJ-rTPJ and rTPJ-mPFC.

### 2.6 Statistical analysis

In a first step, all biomarkers were analysed separately and statistical analysis was conducted to understand: 1) which biomarkers show the strongest differences between AD and controls; and 2) the relative influence of aging and AD status on biomarker values. More precisely, Welch's two-sample t-tests were used to compare differences between groups. Secondly, a General Linear Model with age and group (i.e. AD-status) as factors was used to analyze the associations between each biomarker and these factors. No associations were detected between sex and any of the collected features, and therefore sex was not included in the generalized linear model (GLM) analysis. P-values associated with the GLM were determined using ANOVA.

All p-values were adjusted for multiple comparisons (80 features) using the Benjamin-Hochberg approach [56]. The described analysis was conducted in R (version 3.6) [57].

### 2.7 Classification performance

On the second step, the value of each biomarker separately and in groups to differentiate AD patients from controls was evaluated. Moreover, considering that hippocampal volumes are the main imaging criteria used in the diagnosis of AD, we aimed at understanding whether including additional features could assist the differentiation between AD subjects and healthy controls.

To assess the classification performance of each biomarker individually, multiple logistic regression classifiers were trained using each 2-input features: the biomarker being tested and age. For the analysis of biomarker groups, random forest (RF) [58] classifiers were trained with different sets of features: brain volume features, hippocampal features, diffusion features, structural connectivity features, rsfMRI features, and all features combined. Age was also included in all feature groups. Here, we refer the “structural connectivity features” as the structural connectivity between lobes and hippocampi, and “rsfMRI features” as the functional connectivity in the DMN. We chose a higher spatial resolution of the networks compared with connectomics from the literature, in order to limit the number of features (n = 80) due to small sample size.

Training and Testing of the classifiers were performed following a nested cross-validation scheme where the dataset was divided into 10 subsets with 4 examples each. Then at each time, one of those subsets was excluded and the performance evaluated using the remaining dataset, following 9-fold cross-validation. This allowed assessing not only the mean performance of each classifier but its variability.

Besides the sets of features mentioned above, an additional subset of features was tested containing age and the best feature from each group (hippocampal, brain volumes, diffusion, structural connectivity and rsfMRI). The selection of the best feature per group was made based on the feature importance ranking provided by the “Mean Decrease in Accuracy” metric. This metric is intrinsic to the random forest algorithm and uses the Out-Of-Bag sample of each tree for assessing the performance drop that occurs when the values of the feature being tested are shuffled between examples. The more important features are, the more they are used in the different nodes of a tree and, therefore, the greater the accuracy drop when their values change.

## 3 Results

### 3.1 Statistical analysis

**Figure 2:**
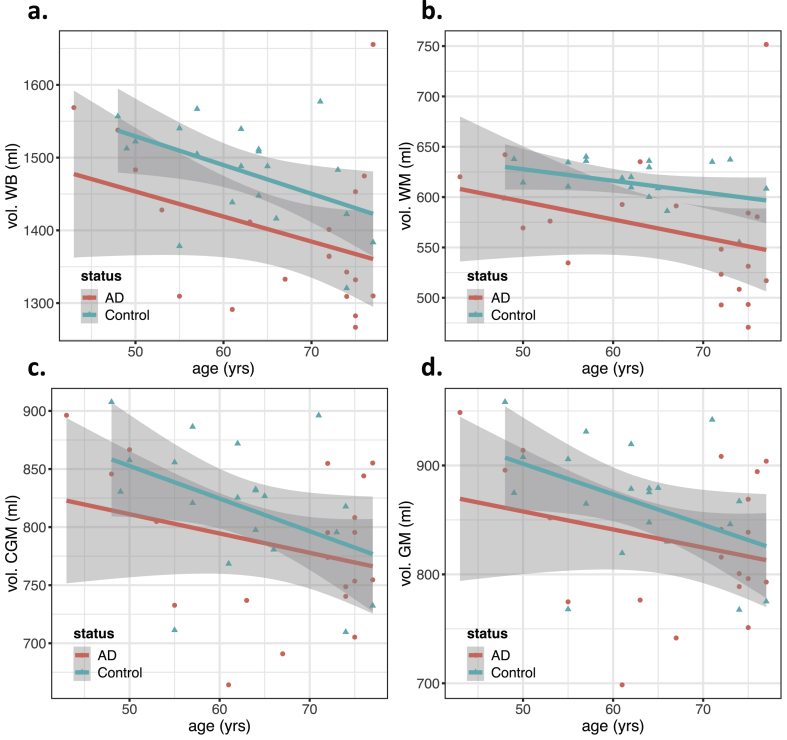
Scatter plots showing brain volumetry features as a function of age, grouped by status (AD/Control). Regression lines for each group are also included, with the gray area showing the 95% confidence intervals. This figure includes the plots for the volumes of a) whole brain (WB), b) white matter (WM), c) cortical gray matter (CGM) and d) gray matter (GM). All volumes were normalized to the same head size, using a reference atlas with an intracranial volume of 1990 ml. Features with significant associations with the subject's group (AD/Control) are marked with an asterisk (see ANOVA results from Table 2).

**Figure 3:**
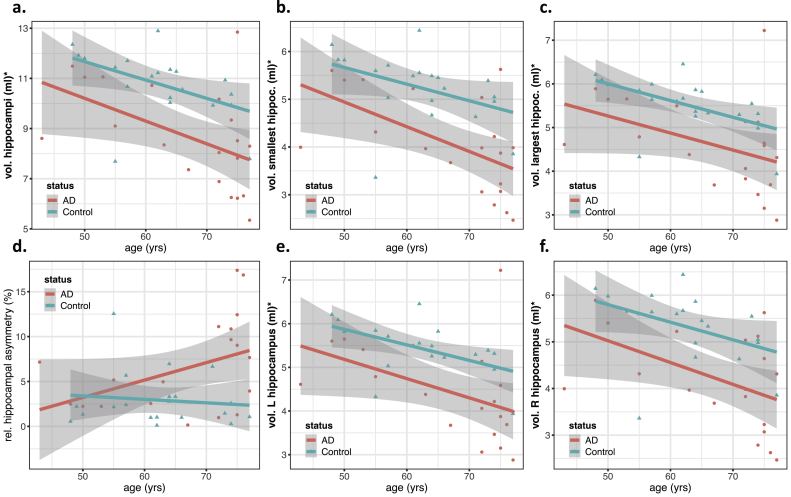
Scatter plots showing hippocampal volumetric features as a function of age, grouped by status (AD/Control). Regression lines for each group are also included, with the gray area showing the 95% confidence intervals. This figure includes the plots for a) the total hippocampal volume, the volumes of the b) smallest and c) largest hippocampus, d) the relative hippocampal asymmetry and the volumes of e) the left and f) right hippocampus in each patient. All volumes were normalized to the same head size, using a reference atlas with an intracranial volume of 1990 ml. Features with significant associations with the subject's group (AD/Control) are marked with an asterisk (see ANOVA results from Table 2).

**Figure 4:**
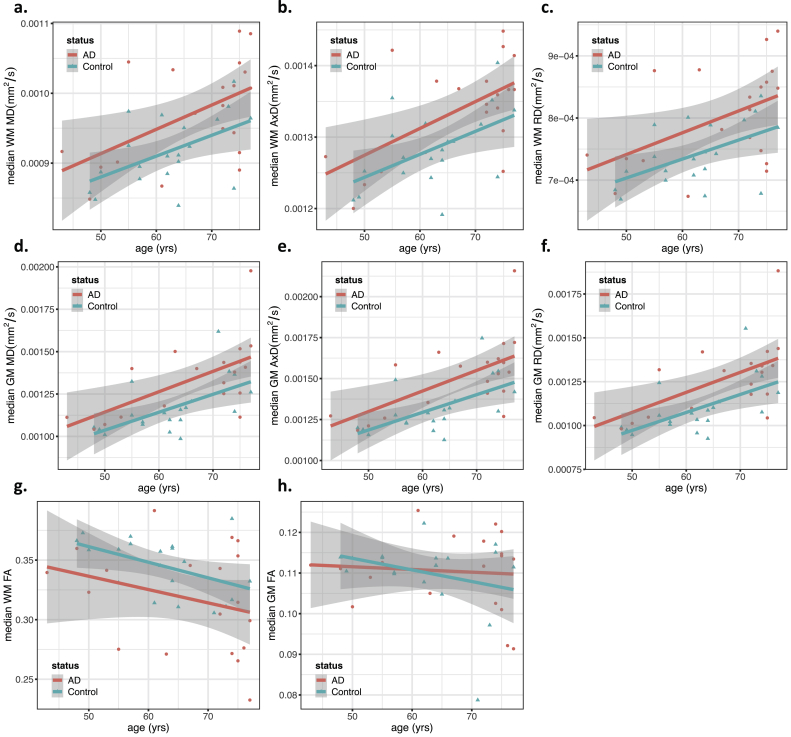
Scatter plots showing each feature as a function of age, grouped by status (AD/Control). Regression lines for each group are also included, with the gray area showing the 95% confidence intervals. This figure includes the plots for the median values in white matter (WM) of a) Mean Diffusivity (MD), b) Axial Diffusivity (AxD), c) Radial Diffusivity (RD), and g) Fractional Anisotropy (FA) and in gray matter (GM) of d) MD, e) AxD, f) RD and h) FA. Features with significant associations with the subject's group (AD/Control) are marked with an asterisk (see ANOVA results from Table 2).

**Figure 5:**
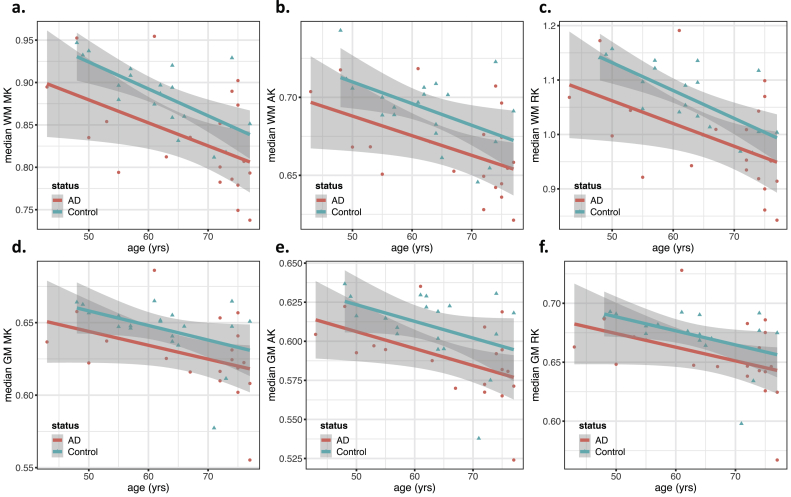
Scatter plots showing diffusion kurtosis features as a function of age, grouped by status (AD/Control). Regression lines for each group are also included, with the gray area showing the 95% confidence intervals. This figure includes the plots for the median values in white-matter (WM), of a) Mean Kurtosis (MK), b) Axial Kurtosis (AK), and c) Radial Kurtosis (RK) and in gray matter (GM) of d) MK, e) AK and f) RK. Features with significant associations with the subject's group (AD/Control) are marked with an asterisk (see ANOVA results from Table 2).

**Figure 6:**
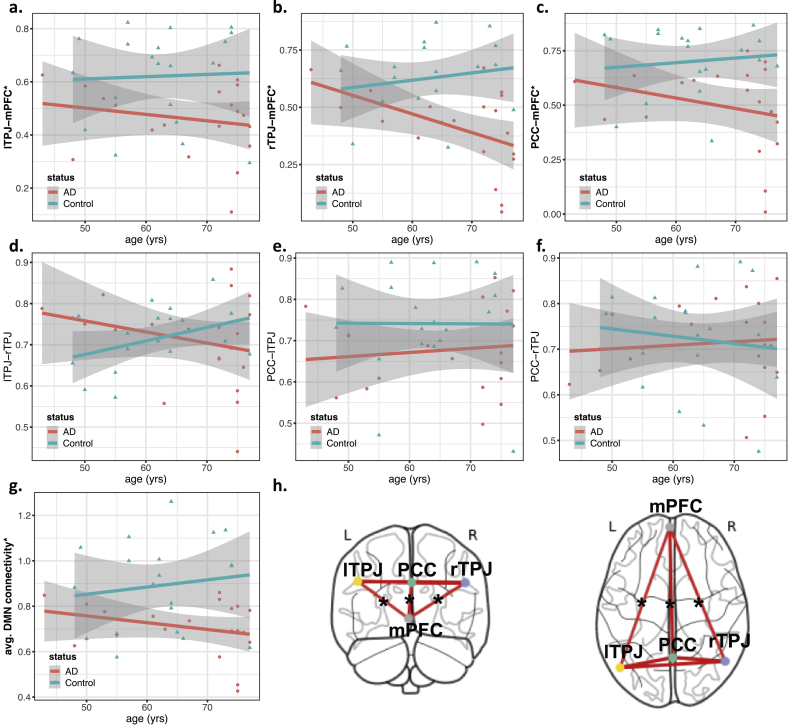
Scatter plots showing functional connectivity features as a function of age, grouped by status (AD/Control). Regression lines for each group are also included, with the gray area showing the 95% confidence intervals. This figure includes the plots for the connectivity between pairs of regions: a) left Temporo-Parietal Junction (lTPJ) and medial Prefrontal Cortex (mPFC), b) right Temporo-Parietal Junction (rTPJ) and mPFC, c) Posterior Cingulate Cortex (PCC) and mPFC, d) lTPJ and rTPJ, e) PCC and lTPJ, f) PCC and rTPJ. The plots of g) the average default mode network (DMN) connectivity and h) a glass-brain representation of the DMN nodes highlighting the connections significantly different between AD and controls are also included. In plots a) to g), features with significant associations with the subject's group (AD/Control) are marked with an asterisk (see ANOVA results from Table 2).

**Figure 7:**
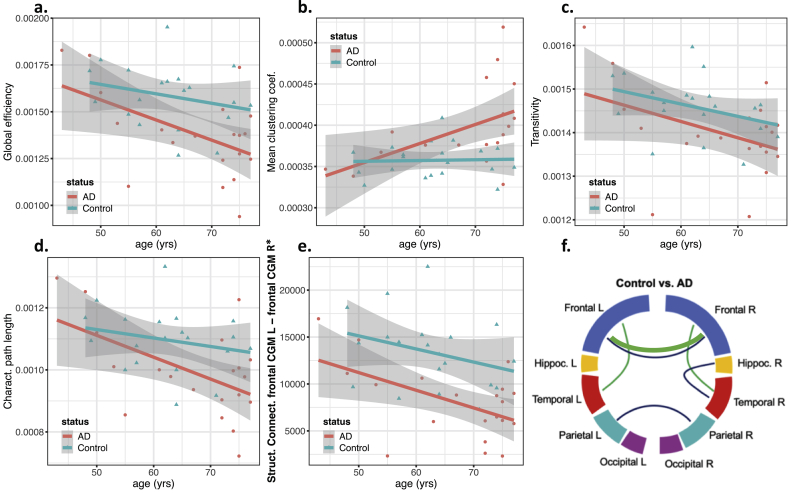
Scatter plots showing structural network features and the most important structural connectivity feature as a function of age, grouped by status (AD/Control). Regression lines for each group are also included, with the gray area showing the 95% confidence intervals. This figure includes a) the global efficiency, b) mean clustering coefficient, c) transitivity, d) characteristic path length, e) structural connectivity between frontal cortical gray matter in the left and right hemisphere (frontal CGM L – frontal CGM R) and f) the representative plot showing the structural connectivity features with significant associations with the subject's AD-status (blue lines) and significant associations with age (green lines) (see ANOVA results from Table 2).

Welch's t-test and ANOVA results are included in Table 2, where only features with significant results were included. These results are supported by Figures 2, 3, 4, 5, 6, and 7, showing the scatter plots of each feature as a function of age. Due to the large number of structural connectivity features, only the scatter plot for the most relevant feature was included and a representative plot showing the significantly affected connections is shown to summarize the results. The ANOVA results of Table 2 for each feature include, as factors, the age and the disease status (AD/Control) of each subject. Sex was not included in the GLM since no feature showed a significant association with sex.

**Table 2:** Combined table of the results of the Welch's t-tests and ANOVA (with group and age as covariates). All p-values were adjusted for 80 multiple comparisons using the Benjamin-Hochberg approach [56]. Significant results (p < 0.05) are marked in bold. Only significant results for the ANOVA test are shown. Full results can be found in appendix.

		Welch's t-test		ANOVA			
				Group (AD/Control)		Age	
		t-statistic	p-val (adjusted)	f-statistic	p-val (adjusted)	f-statistic	p-val (adjusted)
rsfMRI	PCC-mPFC	**3.60**	**0.012**	**11.06**	**0.023**	0.58	0.643
	lTPJ-mPFC	**3.25**	**0.018**	**9.22**	**0.035**	0.21	0.789
	rTPJ-mPFC	**3.91**	**0.010**	**12.66**	**0.023**	1.99	0.345
	avg. DMN connectivity	**3.55**	**0.012**	**11.36**	**0.023**	0.06	0.848
Brain volumes	vol. WB	**2.99**	**0.021**	6.58	0.053	**7.19**	**0.039**
	vol. WM	**3.05**	**0.021**	7.11	0.050	3.94	0.132
Hippoc.	vol. left hippoc.	**3.57**	**0.012**	**10.21**	**0.029**	**10.26**	**0.018**
	vol. right hippoc.	**3.72**	**0.011**	**11.24**	**0.023**	**10.19**	**0.018**
	vol. smallest hippoc.	**4.12**	**0.009**	**14.67**	**0.019**	**12.83**	**0.010**
	vol. largest hippoc.	**3.36**	**0.017**	**8.76**	**0.039**	**9.25**	**0.023**
	total vol. hippocampi	**3.83**	**0.011**	**12.15**	**0.023**	**11.57**	**0.012**
	hippoc. rel. asymmetry	**-2.61**	**0.037**	5.09	0.080	2.38	0.284
dMRI	median WM FA	**2.50**	**0.045**	4.33	0.111	4.89	0.091
	median WM MD	**-2.77**	**0.028**	5.39	0.077	**14.07**	**0.007**
	median WM RD	**-2.76**	**0.028**	5.30	0.077	**11.89**	**0.011**
	median WM AxD	**-2.73**	**0.028**	5.22	0.078	**17.47**	**0.003**
	median WM MK	**3.08**	**0.021**	7.17	0.050	**16.04**	**0.003**
	median WM RK	**2.98**	**0.021**	6.59	0.053	**16.46**	**0.003**
	median WM AK	**3.10**	**0.021**	7.22	0.053	**12.05**	**0.011**
	median GM MD	**-2.95**	**0.021**	6.54	0.053	**21.00**	**0.002**
	median GM RD	**-2.88**	**0.023**	6.12	0.058	**20.32**	**0.002**
	median GM AxD	**-3.05**	**0.021**	7.23	0.050	**21.21**	**0.002**
	median GM MK	2.37	0.055	3.63	0.144	**7.46**	**0.036**
	median GM RK	2.15	0.082	2.80	0.206	**7.95**	**0.032**
	median GM AK	**2.97**	**0.021**	6.44	0.053	**9.56**	**0.022**
Struct. network	global efficiency	**3.10**	**0.021**	7.15	0.050	**7.81**	**0.033**
	charact. Length path	**2.55**	**0.040**	4.37	0.111	**8.01**	**0.032**
	mean clustering coef.	**-2.99**	**0.021**	6.58	0.053	6.38	0.051
	transitivity	2.15	0.082	2.84	0.205	**6.73**	**0.045**
Struct. connect.	temporal L – frontal L	1.75	0.156	1.53	0.343	**8.48**	**0.030**
	temporal L – frontal R	**2.46**	**0.047**	4.20	0.115	4.44	0.105
	temporal R – frontal R	1.34	0.284	0.71	0.549	**6.77**	**0.045**
	temporal R – parietal L	**3.15**	**0.021**	7.66	0.050	3.56	0.153
	temporal R – hippoc. R	**2.94**	**0.021**	6.37	0.053	5.56	0.073
	parietal L – parietal R	**3.36**	**0.016**	**9.49**	**0.035**	0.72	0.584
	parietal L – frontal R	**2.59**	**0.037**	5.56	0.073	0.56	0.643
	parietal R – hippoc. R	**2.84**	**0.023**	**8.08**	**0.048**	0.23	0.789
	frontal L – frontal R	**4.27**	**0.009**	**15.42**	**0.019**	**8.26**	**0.031**

Overall, the results show that 33 out of the 80 tested features showed a significant difference between AD and Controls or an association with AD-status. From all the features analyzed, the ones derived from the hippocampi volumes, structural connectivity between left and right frontal lobes (frontal CGM L-frontal CGM R), as well as the rsfMRI features that include the mPFC and the average DMN functional connectivity, showed the strongest differences between AD and the age-matched healthy controls.

Some of the features tested showed only an association with disease status. This is the case for the functional connectivity features that include the mPFC and structural connectivity between the left and right parietal lobes, as well as between the parietal lobe and hippocampus in the right hemisphere. Features related to hippocampal volume showing significant differences between AD subjects and controls were also significantly affected by aging. In these features, where both disease status and age are significant factors, we see that the presence of Alzheimer's accentuates the changes associated with aging.

Regarding the hippocampal features, the results indicate that in AD the left and right hippocampi are affected differently. In most cases, the left hippocampus is the most affected structure. However, this is not always the case, and therefore the volume of the smallest hippocampus is the feature showing the most pronounced differences between AD and controls. Regarding the other structural features analyzed, the results seem to indicate that AD mainly affects the WM. The volume of GM and CGM was not significantly different between AD patients and controls.

Finally, the diffusion features were the ones that had the greatest association with the age of the subjects, namely the diffusivity in GM (MD, RD, AxD). Furthermore, 5 features showed only an association with age and no group differences: median MK and RK in GM, structural network transitivity and intra-hemispheric structural connectivity between the frontal and temporal lobes.

Due to the intrinsic differences of the two statistical tests used some features have what might seem to be discrepant results between ANOVA and Welch's t-test in terms of significance. This can be explained by several factors, namely: the additional degrees of freedom of the GLM, the discrete vs continuous nature of age and AD-status, the hard significance threshold and the non-linear aspect of the Benjamin-Hochberg correction for multiple comparisons.

### 3.2 Automatic classification

**Figure 8:**
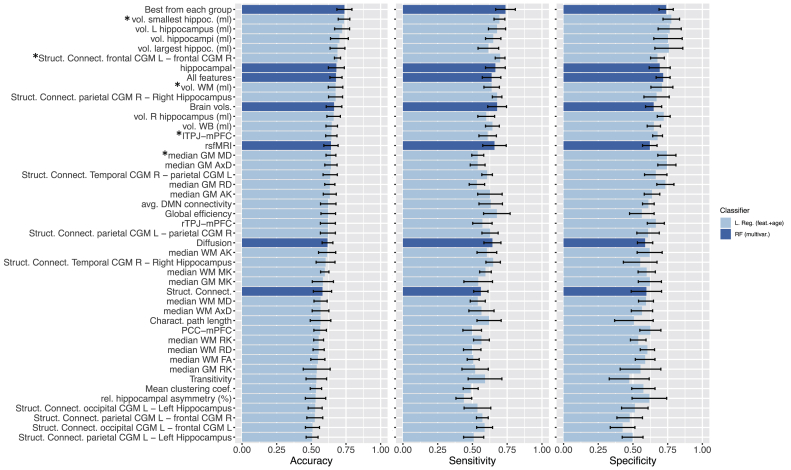
Classification performance for distinguishing AD patients from healthy controls obtained for each feature using univariate logistic regression and for different subsets of features using random forests. Bars show the mean performance values + - SD. Features used in the subset “Best from each group” are marked with an asterisk.

Figure 8 shows the classification performance obtained using logistic regression classifiers trained with each feature together with age, and random forests trained with different subsets of features (all features, brain volumes, hippocampal, rsfMRI, and diffusion features). The results show that the hippocampal features are the features that best discriminate AD patients from controls, confirming their importance in AD diagnosis. From those, the highest discriminative power was obtained by the volume of the smallest hippocampus (mean accuracy: 0.74, mean sensitivity: 0.69, mean specificity: 0.78). The best overall performance was obtained when combining the best features from each group, i.e. smallest hippocampus volume, WM volume, median GM AxD, lTPJ-mPFC functional connectivity, and structural connectivity between frontal CGM L-frontal CGM R. This feature combination achieved a mean accuracy of 0.74, mean sensitivity of 0.74, and specificity of 0.74. For the models trained with all features, the performance was not as high (mean accuracy: 0.68, mean sensitivity: 0.64, mean specificity: 0.72), which might be explained by the noise introduced by the non-informative features combined with the limited size of the dataset used.

Besides the hippocampal features, the WM volume was also an important biomarker to identify AD patients (mean accuracy: 0.68, mean sensitivity: 0.64, mean specificity: 0.71) but not the GM or CGM volumes.

In terms of rsfMRI connectivity features, the lTPJ-mPFC connectivity was the most important feature (mean accuracy: 0.64, mean sensitivity: 0.61, mean specificity: 0.68).

The most discriminative structural connectivity feature was the interhemispheric frontal connectivity (mean accuracy: 0.69, mean sensitivity: 0.70, mean specificity: 0.68) and came just after the hippocampal features in terms of ranking. From the subgroup of network features, the global network efficiency provided the best results but only a mean accuracy of 0.62 was obtained with this feature.

Finally, regarding the global diffusion features, the best performance was obtained with the median GM MD with a mean accuracy of 0.64.

## 4 Discussion

In this work, we replicated previous findings on volumetric and connectivity features for measuring changes due to AD on an independent dataset. Structural and functional brain changes caused by AD seem to be reflected in many of the biomarkers analyzed. Our contribution was to provide a clear overview of the value of multiple structural and connectivity features for diagnosing AD and differentiating AD from healthy aging. In this context, our results showed that differences in structural connectivity (between left and right temporal lobes and between the right hippocampus and right temporal lobe) and functional connectivity (in the DMN) seem to be related only to the disease status and not the age of the subjects, in contrast to the hippocampal features. Then, feature ranking analysis demonstrated that the most promising biomarkers seem to be: the volume of the smallest hippocampus, WM volume, median GM AxD, structural interhemispheric frontal connectivity and functional lTPJ-mPFC connectivity.

At present, clinical use of MRI in AD is mostly focused on the assessment of brain atrophy, namely in the hippocampus [5, 6]. However, brain changes in AD extend well beyond the hippocampal region. Overall, compared to healthy controls, AD patients showed reduced functional brain connectivity, namely between mPFC and the posterior regions of the DMN, reduced interhemispheric connectivity specially in the frontal lobe, reduced WM volume, smaller and more asymmetric hippocampi, higher diffusivity in WM and GM and reduced diffusion kurtosis in both WM and GM.

In terms of structural differences, this study confirms the importance of the hippocampal volumes in AD, a well-established biomarker of this disorder [1, 2]. Interestingly, among the different evaluated features, hippocampal asymmetry was the least relevant. As indicated by the meta-analyses performed by Feng Shi et al. [59], hippocampal asymmetry is observed in a similar extent for healthy controls and AD, while it is more accentuated in Mild Cognitive Impairment (MCI). This seems to suggest that in the prodromal state of AD, atrophy is mostly lateralized but as the disease progresses both the left and right hippocampi are affected, although our results showed increased hippocampal asymmetry over age.

Regarding WM and GM volume differences in AD, previous studies [60] showed as well that the differences in WM volume between AD and controls are more accentuated than GM volume differences. Nevertheless, in the work of Guo et al. significant total GM volume differences were measured. Moreover, VBM analysis showed GM atrophy is mostly localized in the middle and superior temporal gyrus, superior frontal gyrus, inferior parietal lobule, parahippocampal gyrus, insula, caudate head, and thalamus. This suggests that CGM parcellation together with the segmentation of deep gray matter structures may be essential to retrieve discriminant GM atrophy features.

The results obtained with the DMN connectivity biomarkers derived from rsfMRI are in line with previous studies [26, 27, 28, 29, 30, 61], supporting the idea that AD is a disconnection syndrome [4]. However, for introduction of rsfMRI in clinical practice, different challenges in terms of physiological noise reduction, standardization and training need to be addressed [62].

In terms of the diffusion biomarker, the elevated Gaussian diffusivity (MD, AxD, RD) and decreased Kurtosis (MK, AK, RK) seen in WM, indicate a loss of structure in WM and reflect neurodegeneration compatible with AD. Despite the differences in diffusivity parameters, WM FA was less pronounced relative to the other diffusivity parameters. These results are in agreement with the previous literature [63, 64] and reflect the fact that *both* axial and radial diffusivity show increased values in AD. Moreover, the reduced kurtosis values in AD patients, although not specific, may reflect reduced compartmentalization (i.e. tissue complexity) as a result of myelin breakdown and/or axonal degeneration.

In terms of structural connectivity, our results showed reduced connectivity between left and right lobes in frontal and parietal regions. This is in line with a previous study also showing left and right dysconnectivity in patients with AD compared with healthy controls [35]. Disconnection of frontal and parietal areas has been shown to contribute to impaired attention in early AD [65]. Reduced efficiency of the structural network has been demonstrated in patients with AD, which we could reproduce in our study. The reduced global efficiency has been linked to performance in both memory and executive tasks [66, 67].

In terms of classification performance, the results obtained are in line with the literature [68] and show the importance of feature selection, namely in situations where the number of features is in the same order as the number of examples [69]. A higher classification performance would be expected with a larger training dataset and possibly if additionally FDG-PET biomarkers would be used [68]. For this particular dataset, only a very small improvement in performance was obtained when combining multiple features from different modalities in comparison to the classifier trained just with the volume of the smallest hippocampus and age. Nevertheless, the use of additional biomarkers from rsfMRI and DTI may not only be important to discriminate AD from healthy aging but also to differentiate AD from other types of dementia, such as frontotemporal dementia [70] or vascular dementia [71].

One of the main limitations of the current study is the small sample size. This had an impact on the number of connectivity features that could be explored. More precisely, rsfMRI features were restricted to the DMN and the structural network analysis used a coarse CGM parcellation. In addition, only a minor increase in performance was obtained when comparing the use of hippocampus volume alone with the combination of the best features from each group. Similarly to the poor performance obtained with the Random Forest trained with all features, this should come mainly as a result of the limited size of the dataset used here. We believe however that our results on a small sample size are still valid as we could reproduce previous findings from the literature, especially those obtained on large datasets such as ADNI on brain connectivity [32, 33, 34, 35]. Another limitation is that sex distribution of our AD group (40% female subjects) is not representative of the distribution found in the general AD population (around 2/3 of the AD population are women). This, in combination with the sample size, could explain why we did not find any associations between sex and any of the collected features. Finally, we limited our study in the discrimination between patients with AD and healthy controls, without including MCI patients. Previous studies have already demonstrated that DTI and rsfMRI features helped also in detecting patients with MCI [72]. Nevertheless, neuroimaging is currently used in clinical practice to confirm the diagnosis of AD in patients with suspected neurodegenerative disorders. Because our motivation was to determine if DTI and rsfMRI biomarkers would be relevant for clinical use, we did not validate their relevance for patients with MCI. Future research could perform similar analysis as ours on a larger dataset, including MCI patients, for early diagnosis applications that would become essential once disease-modifying treatment will be available.

## 5 Conclusion

Clinical use of MRI in AD is currently restricted to the assessment of brain atrophy. However, inclusion of structural and functional connectivity biomarkers might contribute to a higher diagnostic accuracy and may provide a more complete picture of the clinical status of each patient. From the biomarkers evaluated, some of the most promising to be used together with hippocampal atrophy are: WM volume, structural interhemispheric frontal connectivity, median GM AxD and functional lTPJ-mPFC connectivity.

## Ethics approval

All procedures performed in studies involving human participants were in accordance with the ethical standardsof the institutional and/or national research ethics committee of King Faisal Specialist Hospital & Research Centre (REF:C380/1100/41) and with the 1964 Helsinki declaration and its later amendments or comparable ethical standards.

## Informed consent

Informed consent was obtained from all individual participants included in the study.

## Declarations

### Author contribution statement

R. Mohtasib: Conceived and designed the experiments.

J. Alghamdi, A. Jobeir, A. Masawi, W. Van Hecke, A. Ribbens: Contributed reagents, materials, analysis tools or data.

N. Pedrosa de Barros, T. Billiet, H. Struyfs, T. V. Phan: 2,3,5 Performed the experiments; Analyzed and interpreted the data; Wrote the paper.

### Funding statement

This work was supported by the 10.13039/501100004919King Abdulaziz City for Science and Technology, Saudi Arabia (13-MED1279-20).

### Data availability statement

The authors do not have permission to share data.

### Declarations of interests statement

The authors declare no conflict of interest. N. Pedrosa de Barros, T. Billiet, T. V. Phan, W. Van Hecke, and A. Ribbens are employed by Icometrix.

### Additional information

No additional information is available for this paper.

## Appendix A Supplementary data

Appendix-Full Table 2 Appendix-Full Table 2
