# Supplementary material for: MRI biomarkers for Alzheimer's disease: the impact of functional connectivity in the default mode network and structural connectivity between lobes on diagnostic accuracy
Source: Heliyon. 2022 Feb 2;8(2):e08901. doi: 10.1016/j.heliyon.2022.e08901 (PMC8841367; doi:10.1016/j.heliyon.2022.e08901)
Supplement: Appendix-Full Table 2 [file mmc1.docx]

**Appendix – Full Table 2.** *Combined table of the results of the Welch’s t-tests and ANOVA (with group and age as covariates) for each feature. All p-values were adjusted for 80 multiple comparisons using the Benjamin-Hochberg approach* (56)*. Significant results (p<0.05) are marked in bold.*

|  |  | Welch's t-test | | ANOVA | | | |
| --- | --- | --- | --- | --- | --- | --- | --- |
|  |  |  |  | Group (AD/Control) | | Age | |
|  |  | t-statistic | p-val (adjusted) | f-statistic | p-val (adjusted) | f-statistic | p-val (adjusted) |
| rsfMRI | PCC-lTPJ | 1.79 | 0.147 | 3.23 | 0.174 | 0.10 | 0.829 |
|  | PCC-mPFC | **3.60** | **0.012** | **11.06** | **0.023** | 0.58 | 0.643 |
|  | PCC-rTPJ | 0.33 | 0.812 | 0.09 | 0.813 | 0.01 | 0.952 |
|  | lTPJ-mPFC | **3.25** | **0.018** | **9.22** | **0.035** | 0.21 | 0.789 |
|  | lTPJ-rTPJ | 0.14 | 0.903 | 0.00 | 0.956 | 0.08 | 0.829 |
|  | rTPJ-mPFC | **3.91** | **0.010** | **12.66** | **0.023** | 1.99 | 0.345 |
|  | avg. DMN connectivity | **3.55** | **0.012** | **11.36** | **0.023** | 0.06 | 0.848 |
| Brain volumes | vol. CGM | 1.81 | 0.147 | 1.87 | 0.290 | 4.99 | 0.092 |
|  | vol. GM | 1.89 | 0.126 | 2.14 | 0.264 | 4.75 | 0.095 |
|  | vol. WB | **2.99** | **0.021** | 6.58 | 0.053 | **7.19** | **0.039** |
|  | vol. WM | **3.06** | **0.021** | 7.11 | 0.050 | 3.94 | 0.132 |
| Hippoc. | vol. left hippoc. | **3.57** | **0.012** | **10.21** | **0.029** | **10.26** | **0.018** |
|  | vol. right hippoc. | **3.72** | **0.011** | **11.24** | **0.023** | **10.19** | **0.018** |
|  | rel. hippoc. asymmetry | **-2.61** | **0.037** | 5.10 | 0.080 | 2.38 | 0.284 |
|  | vol. smallest hippoc. | **4.12** | **0.009** | **14.67** | **0.019** | **12.83** | **0.010** |
|  | vol. largest hippoc. | **3.36** | **0.017** | **8.76** | **0.039** | **9.25** | **0.023** |
|  | total vol. hippocampi | **3.83** | **0.011** | **12.15** | **0.023** | **11.57** | **0.012** |
| dMRI | median WM FA | **2.50** | **0.045** | 4.33 | 0.111 | 4.89 | 0.092 |
|  | median WM MD | **-2.77** | **0.028** | 5.39 | 0.077 | **14.07** | **0.007** |
|  | median WM RD | **-2.76** | **0.028** | 5.30 | 0.077 | **11.89** | **0.011** |
|  | median WM AxD | **-2.73** | **0.028** | 5.22 | 0.078 | **17.47** | **0.003** |
|  | median WM MK | **3.08** | **0.021** | 7.17 | 0.050 | **16.04** | **0.003** |
|  | median WM RK | **2.98** | **0.021** | 6.59 | 0.053 | **16.46** | **0.003** |
|  | median WM AK | **3.10** | **0.021** | 7.22 | 0.053 | **12.05** | **0.011** |
|  | median GM FA | -0.12 | 0.903 | 0.11 | 0.810 | 1.02 | 0.531 |
|  | median GM MD | **-2.95** | **0.021** | 6.54 | 0.053 | **21.00** | **0.002** |
|  | median GM RD | **-2.88** | **0.023** | 6.12 | 0.058 | **20.32** | **0.002** |
|  | median GM AxD | **-3.05** | **0.021** | 7.23 | 0.050 | **21.21** | **0.002** |
|  | median GM MK | 2.37 | 0.055 | 3.63 | 0.144 | **7.46** | **0.036** |
|  | median GM RK | 2.15 | 0.082 | 2.80 | 0.206 | **7.95** | **0.032** |
|  | median GM AK | **2.97** | **0.021** | 6.44 | 0.053 | **9.56** | **0.022** |
| Structural network | Global efficiency | **3.10** | **0.021** | 7.15 | 0.050 | **7.81** | **0.033** |
|  | Mean clustering coef. | **-2.99** | **0.021** | 6.48 | 0.053 | 6.38 | 0.051 |
|  | Transitivity | 2.15 | 0.082 | 2.85 | 0.205 | 7.73 | 0.051 |
|  | Charact. Length path | **2.55** | **0.040** | 4.37 | 0.111 | **8.013** | **0.032** |

|  |  | Welch's t-test | | ANOVA | | | |
| --- | --- | --- | --- | --- | --- | --- | --- |
|  |  |  |  | Group (AD/Control) | | Age | |
|  |  | t-statistic | p-val (adjusted) | f-statistic | p-val (adjusted) | f-statistic | p-val (adjusted) |
| Struct. connect. | temporal L – temporal R | 1.32 | 0.285 | 1.12 | 0.436 | 1.10 | 0.51 |
|  | temporal L – parietal L | 1.07 | 0.390 | 0.56 | 0.610 | 1.98 | 0.345 |
|  | temporal L – parietal R | 1.89 | 0.126 | 2.14 | 0.264 | 4.68 | 0.096 |
|  | temporal L – occipital L | 0.13 | 0.903 | 4.68 e-05 | 0.995 | 0.30 | 0.789 |
|  | temporal L – occipital R | 0.14 | 0.903 | 0.02 | 0.913 | 1.67 | 0.379 |
|  | temporal L – frontal L | 1.75 | 0.156 | 1.53 | 0.343 | **8.48** | **0.030** |
|  | temporal L – frontal R | **2.46** | **0.047** | 4.20 | 0.115 | 4.44 | 0.105 |
|  | temporal L – hippoc. L | 1.33 | 0.285 | 0.86 | 0.496 | 3.56 | 0.153 |
|  | temporal L – hippoc. R | 0.73 | 0.586 | 0.26 | 0.741 | 0.86 | 0.544 |
|  | temporal R – parietal L | **3.15** | **0.021** | 7.66 | 0.050 | 3.56 | 0.153 |
|  | temporal R – parietal R | 0.65 | 0.622 | 0.12 | 0.810 | 1.83 | 0.361 |
|  | temporal R – occipital L | 0.43 | 0.763 | 0.12 | 0.810 | 0.09 | 0.829 |
|  | temporal R – occipital R | -0.26 | 0.837 | 0.19 | 0.779 | 0.73 | 0.584 |
|  | temporal R – frontal L | 1.18 | 0.332 | 0.53 | 0.616 | 4.95 | 0.092 |
|  | temporal R – frontal R | 1.34 | 0.284 | 0.71 | 0.549 | **6.77** | **0.045** |
|  | temporal R – hippoc. L | 1.73 | 0.159 | 2.16 | 0.264 | 0.95 | 0.540 |
|  | temporal R – hippoc. R | **2.94** | **0.021** | 6.37 | 0.053 | 5.56 | 0.073 |
|  | parietal L – parietal R | **3.36** | **0.016** | **9.49** | **0.035** | 0.72 | 0.584 |
|  | parietal L – occipital L | 0.28 | 0.837 | 0.11 | 0.810 | 0.10 | 0.829 |
|  | parietal L – occipital R | 0.34 | 0.736 | 0.07 | 0.827 | 0.08 | 0.829 |
|  | parietal L – frontal L | -0.38 | 0.798 | 0.36 | 0.682 | 1.12 | 0.511 |
|  | parietal L – frontal R | **2.60** | **0.037** | 5.56 | 0.073 | 0.56 | 0.643 |
|  | parietal L – hippoc. L | 2.21 | 0.077 | 4.10 | 0.12 | 0.27 | 0.789 |
|  | parietal L – hippoc. R | 1.34 | 0.284 | 1.14 | 0.436 | 1.33 | 0.467 |
|  | parietal R – occipital L | 1.02 | 0.412 | 0.96 | 0.466 | 0.0004 | 0.984 |
|  | parietal R – occipital R | -0.91 | 0.475 | 0.46 | 0.638 | 0.95 | 0.540 |
|  | parietal R – frontal L | 0.69 | 0.597 | 0.49 | 0.629 | 0.02 | 0.914 |
|  | parietal R – frontal R | -1.30 | 0.291 | 1.80 | 0.295 | 0.16 | 0.810 |
|  | parietal R – hippoc. L | 1.19 | 0.332 | 1.08 | 0.436 | 0.27 | 0.786 |
|  | parietal R – hippoc. R | **2.84** | **0.023** | **8.08** | **0.048** | 0.23 | 0.789 |
|  | occipital L – occipital R | -1.99 | 0.119 | 2.64 | 0.220 | 2.81 | 0.227 |
|  | occipital L – frontal L | 2.15 | 0.082 | 3.88 | 0.129 | 0.25 | 0.789 |
|  | occipital L – frontal R | 1.67 | 0.170 | 1.86 | 0.290 | 1.76 | 0.368 |
|  | occipital L – hippoc. L | 1.92 | 0.126 | 3.01 | 0.192 | 0.34 | 0.779 |
|  | occipital L – hippoc. R | 0.58 | 0.669 | 0.104 | 0.810 | 1.23 | 0.487 |
|  | occipital R – frontal L | 0.72 | 0.586 | 0.248 | 0.742 | 0.88 | 0.544 |
|  | occipital R – frontal R | 1.18 | 0.332 | 1.08 | 0.436 | 0.21 | 0.789 |
|  | occipital R -hippoc. L | 1.65 | 0.176 | 2.28 | 0.264 | 0.14 | 0.810 |
|  | occipital R – hippoc. R | 1.79 | 0.147 | 2.17 | 0.264 | 1.86 | 0.361 |
|  | frontal L – frontal R | **4.27** | **0.009** | **15.42** | **0.019** | 8.26 | 0.031 |
|  | frontal L – hippoc. L | 1.55 | 0.202 | 2.01 | 0.275 | 0.15 | 0.810 |
|  | frontal L – hippoc. R | -0.84 | 0.519 | 0.37 | 0.682 | 0.92 | 0.541 |
|  | frontal R – hippoc.L | -0.53 | 0.699 | 0.18 | 0.785 | 0.16 | 0.810 |
|  | Frontal R – hippoc. R | 1.60 | 0.187 | 2.10 | 0.264 | 0.21 | 0.789 |
|  | Hippoc. L – Hippoc. R | 0.29 | 0.837 | 0.07 | 0.827 | 0.007 | 0.952 |
